# Aging-associated changes in L-type calcium channels in the left atria of dogs

**DOI:** 10.3892/etm.2013.1266

**Published:** 2013-08-20

**Authors:** TIAN-YI GAN, WEIWEI QIAO, GUO-JUN XU, XIAN-HUI ZHOU, BAO-PENG TANG, JIAN-GUO SONG, YAO-DONG LI, JIAN ZHANG, FA-PENG LI, TING MAO, TAO JIANG

**Affiliations:** 1 Department of Cardiology, The First Affiliated Hospital, Xinjiang Medical University, Urumqi, Xinjiang 830011;; 2 The Key Laboratory of Cardiovascular Remodeling and Function Research, Chinese Ministry of Education and Chinese Ministry of Public Health, Shandong University, Qilu Hospital, Jinan, Shandong 250012;; 3 Department of Cardiology, Yantaishan Hospital, Yantai, Shandong 264001, P.R. China

**Keywords:** atria, calcuim channel, cellular electrophysiology, aging

## Abstract

Action potential (AP) contours vary considerably between the fibers of normal adult and aged left atria. The underlying ionic and molecular mechanisms that mediate these differences remain unknown. The aim of the present study was to investigate whether the L-type calcium current (I_Ca.L_) and the L-type Ca^2+^ channel of the left atria may be altered with age to contribute to atrial fibrillation (AF). Two groups of mongrel dogs (normal adults, 2–2.5 years old and older dogs, >8 years old) were used in this study. The inducibility of AF was quantitated using the cumulative window of vulnerability (WOV). A whole-cell patch-clamp was used to record APs and I_Ca.L_ in left atrial (LA) cells obtained from the two groups of dogs. Protein and mRNA expression levels of the a1C (Cav1.2) subunit of the L-type calcium channel were assessed using western blotting and quantitative PCR (qPCR), respectively. Although the resting potential, AP amplitude and did not differ with age, the plateau potential was more negative and the APD_90_ was longer in the aged cells compared with that in normal adult cells. Aged LA cells exhibited lower peak I_Ca.L_ current densities than normal adult LA cells (P<0.05). In addition, the Cav1.2 mRNA and protein expression levels in LA cells were decreased in the aged group compared with those in the normal adult group. The lower AP plateau potential and the decreased I_Ca.L_ of LA cells in aged dogs may contribute to the slow and discontinuous conduction of the left atria. Furthermore, the reduction of the expression levels of Cav1.2 with age may be the molecular mechanism that mediates the decline in I_Ca.L_ with increasing age.

## Introduction

Aging is known to increase the propensity for the occurrence of atrial arrhythmias, particularly atrial fibrillation (AF) (1,2). AF usually occurs in conjunction with other cardiovascular diseases; however, not all patients with AF have an underlying disease (3). This suggests that age-associated changes in the atrium may be important in the development of AF (4). However, the electrophysiological changes that cause the atria of elderly individuals to be more susceptible to AF than those of younger adults remain poorly understood.

The role of the left atrium in the development of AF has previously been identified (5), and previous studies have shown that in left atrial (LA) tissue, the action potential (AP) duration (APD) is prolonged and the AP plateau becomes increasingly negative with age (6,7). These alterations in the AP may provide a substrate for reentry, which facilitates the occurrence and maintenance of reentrant arrhythmias, including AF (8). The L-type Ca^2+^ current (I_Ca.L_) is the major current determining the plateau level of the AP; thus, variations in I_Ca.L_ may lead to changes in the AP plateau level. Previous studies have reported that I_Ca.L_ is reduced in the right atrial (RA) cells of aged canines compared with that in cells from younger adult canines (9,10). However, to the best of our knowledge, there are few published data on the effects of age on LA I_Ca.L_(7).

The I_Ca.L_ is mediated by the L-type Ca^2+^ channel. The a1C (Cav1.2) subunit is considered to be the most important polypeptide of the Ca^2+^ channel-forming proteins, since it forms the channel pore for ion flow. However, published data concerning the effects of age on LA Cav1.2 expression levels are lacking.

In the present study, we tested the hypothesis that I_Ca.L_ and Cav1.2 expression levels in the left atrium change with age, creating a substrate that favors the initiation of AF. This was achieved by investigating the differences in I_Ca.L_ and Cav1.2 expression in LA myocardia between adult and aged dogs.

## Materials and methods

### Ethics

All experiments conformed to the Guide for the Care and Use of Laboratory Animals published by the US National Institutes of Health (11). All animal studies were approved by the Animal Use and Care Committee of the First Teaching Hospital, Xinjiang Medical University (Urumqi, China).

### Animal preparation

Seven adult (2–2.5 years old) and ten aged (>8 years old) mongrels of either gender, weighing 18–26 kg, were used in this study. The ages of the dogs were estimated by a veterinarian based on standard measures for age, including dentition, coat, eyes and musculoskeletal and conformational descriptors. All dogs were anesthetized with sodium pentobarbital (30 mg/kg) and ventilated with atmospheric air using a positive pressure respirator. Core body temperature was maintained at 36.5±1.5°C. The dogs were subjected to twelve-lead electrocardiograms (ECGs) to confirm sinus rhythm and echocardiograms were performed to exclude structural heart disease. Subsequently, continuous recording of standard ECG leads was carried out to determine the heart rate and rhythm. Blood pressure (BP) was continuously monitored via a pressure transducer positioned in the right femoral artery.

The chest was entered via a left thoracotomy at the 4th intercostal space. Multi-electrode catheters (Biosense-Webster, Diamond Bar, CA, USA) were secured to allow recording at the LA appendage (LAA), left superior pulmonary vein (LSPV) and left inferior pulmonary vein (LIPV). Similar electrode catheters were attached to the RA appendage (RAA), right superior pulmonary vein (RSPV) and right inferior pulmonary vein (RIPV) via a right thoracotomy at the 4th intercostal space. All traces from the electrode catheters were amplified and digitally recorded using a computer-based Lab System (GE 2000; General Electric Company, Fairfield, CT, USA). Bipolar electrograms were filtered at 30–500 Hz. ECG filter settings were 0.1–250 Hz.

### Induction of AF

Rapid atrial pacing was delivered (1,000 bpm; 2X threshold; duration, 1 msec) at the RAA. After 30 min, rapid atrial pacing was terminated in order to measure AF inducibility. Programmed stimulation at atrial myocardial sites or pulmonary vein (PV) sleeves was performed using a programmable stimulator (DF-4A; Suzhou Dongfang Electronic Instrument Factory, Suzhou, China). Programmed pacing consisted of eight consecutive stimuli (S1–S1, cycle length=330 msec) followed by a premature stimulus (S1–S2) that was progressively decremented until refractoriness. Pacing was performed at 2X diastolic threshold (TH) and at 4X TH. AF was defined as irregular atrial rates (>500 bpm) associated with irregular atrioventricular conduction (lasting >5 sec). The window of vulnerability (WOV) was used as a quantitative measure of AF inducibility. AF inducibility was quantitated as the longest S1–S2 minus the shortest S1–S2 that induced AF at each pacing TH. The cumulative WOV was the sum of the individual WOVs.

### Atrial myocyte preparation

Following intravenous (i.v.) administration of pentobarbital (30 mg/kg) and thoracotomy, the heart was rinsed in oxygenated Ca^2+^-free Tyrode’s solution [137 mmol/l NaCl, 5.4 mmol/l KCl, 1.0 mmol/l MgCl_2_, 0.33 mmol/l NaH_2_PO_4,_, 10 mmol/l HEPES and 10 mmol/l glucose (adjusted to pH 7.4 with NaOH)]. The aorta was cannulated and the heart was retrogradely perfused on a Langendorff apparatus (ADInstruments, Inc., New South Wales, Australia) at 37°C. A perfusion with Ca^2+^-free Tyrode’s solution for 5 min was followed by perfusion with Ca^2+^-free Tyrode’s solution containing 0.03% collagenase-II (Worthington Biochemical, Lakewood, NJ, USA) and 1% BSA for 35 min. The left atria were dissected, minced and gently triturated with a pipette in the low-Ca^2+^ Tyrode’s solution containing 1% BSA at 37°C for 10 min. The cells were filtered through 200-*μ*m nylon mesh and resuspended in the Tyrode’s solution, in which the Ca^2+^ concentration was gradually increased to 1.0 mmol/l. Only cells with a rod-shaped morphology and clear cross-striation were used for subsequent experiments.

### Cellular electrophysiology

LA cells were continuously superfused (2–3 ml/min) in a 1-ml bath with normal Tyrode’s solution [137 mmol/l NaCl, 5.4 mmol/l KCl, 1.0 mmol/l MgCl_2_, 1.8 mmol/l CaCl_2_, 0.33 mmol/l NaH_2_PO_4,_, 10 mmol/l HEPES and 10 mmol/l glucose (adjusted to pH 7.4 with NaOH)]. The solution was bubbled with 100% O_2_. Membrane currents and APs were recorded using whole-cell patch-clamp techniques with an EPC 10 Double amplifier and Patchmaster software (HEKA Elektronik Dr. Schulze GmbH, Lambrecht/ Pfalz, Germany). Patch pipette resistances ranged from 2.0–3.0 MΩ when filled with an internal solution. APs were recorded in current-clamp mode. The solution for AP recordings contained 137 mmol/l NaCl, 5.4 mmol/l KCl, 1.0 mmol/l MgCl_2_, 1.8 mmol/l CaCl_2_, 10 mmol/l HEPES and 20 mmol/l glucose (adjusted to pH 7.4 with KOH). The internal electrode solution for AP recordings contained 140 mmol/l KCl, 2.0 mmol/l MgCl_2_, 2.0 mmol/l egtazic acid, 5.0 mmol/l HEPES, 5 mmol/l EGTA and 4.0 mmol/l Na_2_ ATP (adjusted to pH 7.4 with KOH). The Ca^2+^ currents were recorded in voltage-clamp mode. The external solution for I_Ca-L_ recording contained 137 mmol/l choline-Cl, 2.0 mmol/l CaCl_2_, 1.0 mmol/l MgCl_2_, 5 mmol/l HEPES, 10 mmol/l glucose, 4.6 mmol/l CsCl, 10 mmol/l TEA-Cl and 5 mmol/l 4-aminopyridine (4-AP) (adjusted to pH 7.30 with CsOH). The internal solution for I_Ca.L_ recording contained 120 mmol/l CsCl, 1.0 mmol/l MgCl_2_, 5.0 mmol/l MgATP, 10 mmol/l BAPTA, 10 mmol/l HEPES and 10 mmol/l TEA-Cl (adjusted to pH 7.3 with CsOH). Data acquisition was initiated 10 min after membrane rupture. I_Ca.L_ magnitudes were normalized by the membrane capacitance (pF) of each cell and expressed as current density (pA/pF). Recordings were filtered using low-pass (2 Hz) and high-pass (30 Hz) filters.

### Detection of Cav1.2 gene expression

Total RNA was extracted from LAA samples using TRIzol (Gibco-BRL, Carlsbad, CA, USA). Total RNA was reverse transcribed using ReverTra Ace (Toyobo Biotech Co., Ltd., Osaka, Japan). The expression levels of target genes were measured by quantitative PCR (qPCR) using Sybr-Green qPCR Master Mix (Bio-Rad, Hercules, CA, USA). In each assay, β-actin (used as an endogenous control) and Cav1.2 genes from the same samples were amplified in triplicate in separate tubes. The mRNA levels of Cav1.2 were determined using the relative standard curve method, normalized against the corresponding β-actin mRNA levels and then expressed as the relative change from the control ± SD. The expected sizes of amplicons were confirmed by gel electro-phoreses. The sequences of the genes studied were obtained from GenBank and the primers were designed using PRIMER 5.0 software (Applied Biosystems, Carlsbad, CA, USA). The amplicon size and primer sequences for the genes are shown in Table I.

### Assessment of Cav1.2 protein expression

For western blotting, 50 *μ*g protein was solubilized for 5 min at 95°C in one volume of loading buffer, loaded onto 10% SDS-PAGE gels and then transferred to nitrocellulose membranes (Bio-Rad, Hercules, CA, USA). The membranes were blocked with 5% nonfat dry milk in PBST (containing 0.05% Tween 20), incubated overnight at 4°C with the primary antibody (Cav1.2, 1:2,000; goat IgG, polyclone; Santa Cruz Biotechnology Inc., Santa Cruz, CA, USA), washed in PBST, incubated with horseradish peroxidase-conjugated secondary antibody and revealed using Immun-Star HRP Substrate (Bio-Rad). For normalization of gel loading, the same western blots were reprobed with anti-β-actin (dilution, 1:200; Santa Cruz Biotechnology Inc.). The densities of the bands on the western blots were quantified using an automatic gel imaging and analysis system (Bio-Rad).

### Statistical analysis

Statistical analysis was performed using SPSS 18.0 software (SPSS, Inc., Chicago, IL, USA). All values are expressed as the mean ± SD. Comparisons between the two groups were made using the Student’s t test. P<0.05 was considered to indicate a statistically significant difference.

## Results

### ECG data

ECG data concerning the sinus rhythm for the adult and aged groups are shown in Table II. The ECGs of the aged group exhibited prolonged P wave durations and increased P wave dispersion (PWD) compared with those of the adult group. Other variables were not observed to differ between the two groups.

### Induced AF

**Figure 1.**
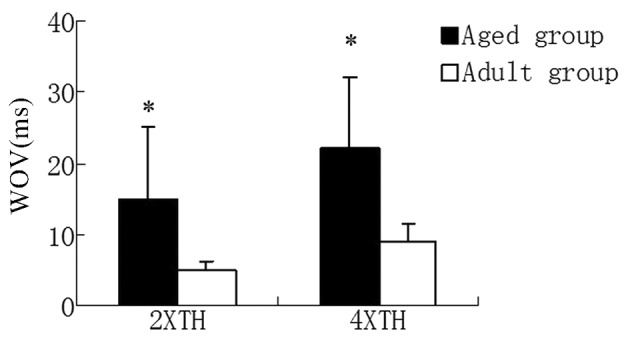
Determination of the window of vulnerability (WOV) for atrial fibrillation (AF) when programmed stimulation was performed at the RAA in adult and aged dogs. ^*^P<0.05 vs. adult dogs. RAA, right atrial appendage; TH, threshold.

As shown in Fig. 1, programmed electrical stimulation at the RAA in the aged group induced a larger WOV (15±7.5 ms) compared with that of the adult group (5±2.5 msec) during 2X TH. A similar result was observed during 4X TH (aged group, 22±12 msec vs. adult group, 9±4.5 msec).

### AP characteristics

**Figure 2.**
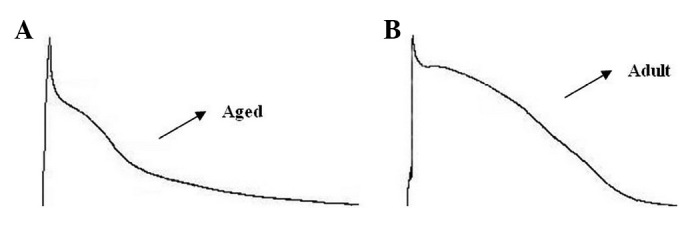
Action potential (AP) recording from the left atrial (LA) cells of (A) aged and (B) adult dogs.

LA cells from aged atria exhibited longer APDs and lower plateau potentials compared with those from adult atria. Representative AP recordings from the adult and aged groups are shown in Fig. 2. AP characteristics, at a cycle length of 2,000 msec, are shown in Table III. While there were no significant differences in the maximum diastolic potential (MDP) or action potential amplitude (APA), action potential duration to 90% repolarization (APD_90_) was longer in the aged dogs, indicating that the slope of phase 3 repolarization was more gradual in the aged than in the adult cells.

### I_Ca.L_

**Figure 3.**
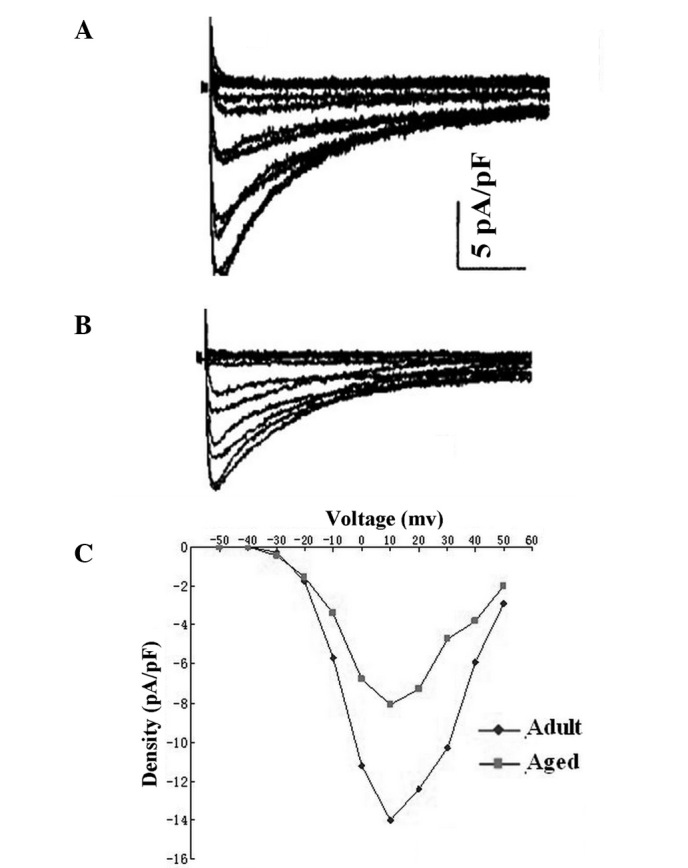
Typical L-type calcium current (I_ca-L_) recordings obtained from left atrial (LA) myocytes in adult and aged dogs. (A) Adult LA cell; (B) aged LA cell; (C) mean I_ca-L_ density-voltage relationship in adult (cells, n=11; hearts, n=7) and aged (cells, n=13; hearts, n=10) LA cells.

Typical I_Ca.L_ recordings from LA cells of the adult and aged dogs are shown in Fig. 3. Aged LA cells had lower peak I_Ca.L_ densities than adult LA cells (−8.1±0.5 vs. −14.1±0.8, respectively, P<0.05; measured at +10 mV). Activation voltage dependence was assessed from depolarization-induced currents and the driving force was corrected with driving force corrected by membrane potential-reversal potential, where reversal potential is the voltage axis intercept of the ascending limb of the current-voltage relation. There were no significant differences in half-activation voltage or slope factor between the two groups (Table IV). Inactivation was assessed with 1-sec prepulses of −60, −50, −40, −30, −20, −10, 0, 10, 20, 30 and 40 mV, followed by 250-msec test pulses to +10 mV. Furthermore, the current reduction in the aged cells was not accompanied by a significant change in the refractory period (Table IV).

### Cav1.2 gene expression in LA cells

**Figure 4.**
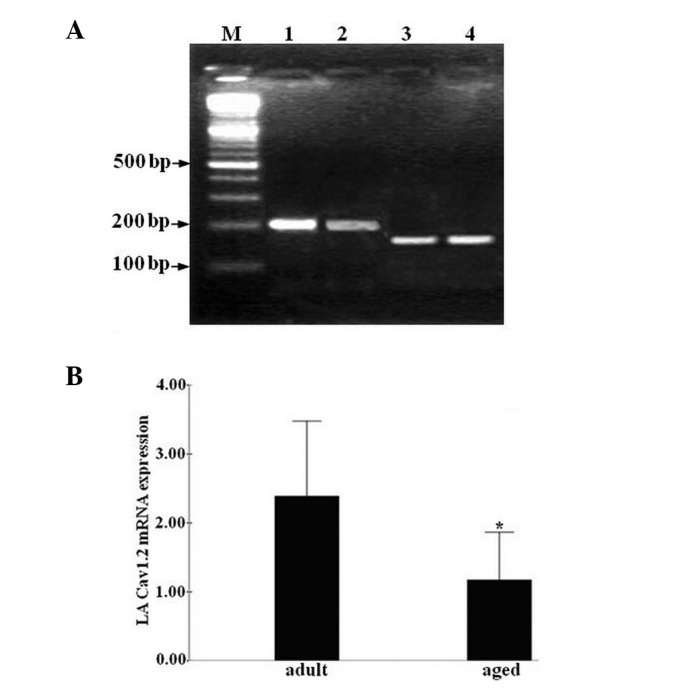
Decline in Cav1.2 mRNA expression with increasing age in the left atrial (LA) cells of canines. (A) Quantitative PCR (qPCR) products for Cav1.2 and β-actin. M, DNA marker; lane 1, Cav1.2 (adult); lane 2, Cav1.2 (aged); lane 3, β-actin (adult); lane 4, β-actin (aged). (B) Relative Cav1.2 mRNA expression levels. ^*^P<0.05 vs. adult canines.

To study Cav1.2 expression in LA cells, Cav1.2 mRNA levels were analyzed using qPCR. The specificity of the amplified PCR product was verified using agarose gel electrophoresis and non-specific DNA fragments were not detected (Fig. 4A). Cav1.2 mRNA levels were decreased in the aged group compared with those in the adult group (P<0.05, Fig. 4B).

### Cav1.2 protein expression in LA cells

**Figure 5.**
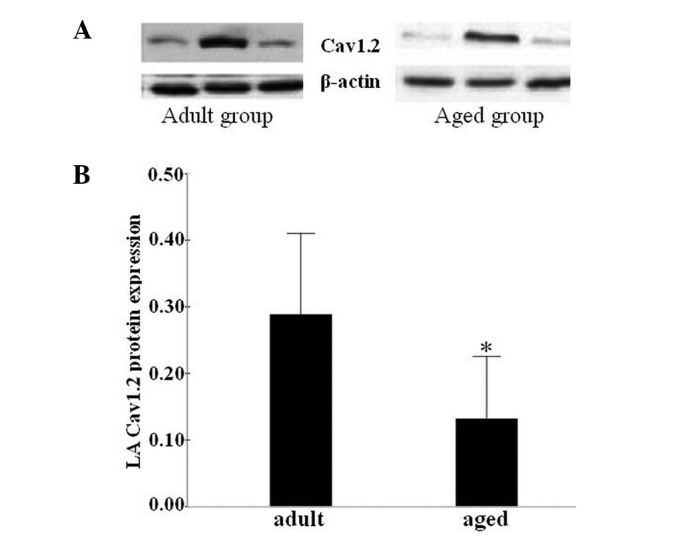
Decline in Cav1.2 protein expression levels with increasing age in the left atrial (LA) cells of dogs. (A) Western blotting bands for Cav1.2 and β-actin. (B) Relative Cav1.2 protein expression levels. ^*^P<0.05 vs. adult dogs.

To confirm the qPCR results, western blotting was performed with Cav1.2 antibodies. Fig. 5A presents the bands from a gel on which the Cav1.2 protein levels from LA cells were studied. The β-actin bands were used to confirm that the loading was equal. Densitometric data demonstrated that the Cav1.2 protein levels were significantly lower in the aged group compared with those in the adult group (P<0.05, Fig. 5B).

## Discussion

In the present study, the susceptibility to AF was greater in the aged dog group than in the adult group. We hypothesized that certain electrophysiological changes in the atria were responsible for this difference. We demonstrated that certain characteristics of the APs in LA cells change with increasing age. The most marked alteration was a significant lowering of the plateau potential in aged LA cells. Previous studies have shown that negative plateau potentials have a lower driving force in the conduction of early premature beats (12). Therefore, our results imply that alterations in the APs of aged atrial cells are likely to lead to a decreased conduction of premature beats in aged atria. The slow conduction of early premature impulses may further facilitate the onset of AF. The currents that determine the plateau level of APs in the atrium are I_Kur_, I_to_ and I_Ca.L_(13). Therefore, a reduction in the depolarizing current (I_Ca.L_) or an increase in the repolarizing currents (I_Kur_ and/or I_to_) may lead to a lower AP plateau (14). Moreover, the APD_90_ value in LA cells was prolonged with age; this may be the result of age-induced changes in the delayed rectifier potassium current (I_K_) or may simply be a consequence of the low AP plateau in aged canines.

In addition to age-associated changes in the I_Ca.L_ of LA cells, previous studies have also reported that I_Ca.L_ is reduced in the RA cells of aged canines compared with those of adult canines. However, no previous studies have reported on the effects of age on LA cell I_Ca.L_. In the current study, we showed that there was a significant reduction in the peak I_Ca.L_ in aged canine LA cells compared with that in adult canine LA cells. The current reduction in aged cells was not accompanied by a significant change in Ca^2+^ channel availability or recovery from inactivation. These results suggest that a reduction in the I_Ca.L_ is a major mechanism underlying the low AP plateau in aged canine LA cells.

The PWD was significantly longer in the aged dogs than in the adult canines. This may be a result of the degree of age-associated reduction in the conduction of the atria (15). Age-related changes in the content and distribution of connective tissue in the atria reduce the degree of cellular coupling and lead to discontinuous propagation, thus slowing conduction (16). Under normal conditions, sufficient depolarizing current is transferred across such discontinuities to maintain normal propagation. However, when the depolarizing current is reduced, conduction slows (17). The driving force of the discontinuous conduction is determined by the plateau potential, and the I_Ca.L_ is important in the maintenance of conduction (18,19). Therefore, the present results imply that the reductions in I_CaL_ and AP plateau potential in aged LA cells may lead to the occurrence of discontinuous conduction. These findings may constitute a mechanism via which aged atria become more susceptible to AF.

In cardiac myocytes, Ca^2+^ currents through L-type Ca^2+^ channels are the main mechanism for Ca^2+^ influx from the extracellular space into the cytoplasm (20). Cardiac L-type Ca^2+^ channels are composed of four polypeptide subunits (α1*,* β*,* α2 and δ) (21). The α1 subunit is the most important polypeptide of the Ca^2+^ channel-forming proteins; it forms the channel pore for ion flow and is responsible for voltage-dependent Ca^2+^ channel opening and channel selectivity for Ca^2+^ ions (22,23). To date, at least 10 different α1 subunit genes have been identified, but only the a1C (Cav1.2) isoform is expressed at high levels in cardiac muscle (24). The current study demonstrated that Cav1.2 mRNA and protein expression levels in LA cells were significantly lower in the aged group compared with those in the adult group. This may be the main cause of the reduction in I_Ca.L_ in aged canines. Jones *et al* (25) obtained similar findings, demonstrating that within the sinoatrial (SA) node, an age-related decline in the expression levels of the Cav1.2 protein caused the suppression of AP formation and propagation, leading to failure of the SA node as a pacemaker. Therefore, atria Cav1.2 protein levels may decrease with age. However, research has been limited to studies using Cav1.2 protein from only one region of the left atrium and SA node (one region of RA). The mechanisms underlying these changes are unknown. We hypothesized that aging may result in the progressive deterioration of physiological functions and metabolic processes, which alters the density and distribution of ion channels.

Previous studies have attached particular importance to the left atrium in the initiation and maintenance of AF (26,27). The present study demonstrated that there were age-associated changes in the electrophysiological properties and ion channels of the LA myocardium. A lower AP plateau potential and decreased I_Ca.L_ in the LA cells of aged canines may contribute to the slow and discontinuous conduction in the left atrium. These changes increase the susceptibility of aged atria to AF. Furthermore, the decreased expression of Cav1.2 with age may be the cause of the reduction in I_Ca.L_ with increasing age. However, further studies of the mechanisms of alteration in Cav1.2 expression are required.

Although the present study demonstrated age-associated electrophysiological and molecular changes in the LA cells of aged canines, the extent to which these effects are clinically applicable remains to be determined.

## Figures and Tables

**Table I.** Amplicon size and primer sequences of genes.

Gene	Primer sequence	Amplicon size (bp)
β-actin	F: 5′-AAGGACCTGTATGCCAACACA-3′	152
	R: 5′-ATCCACACAGAATACTTGCGTT-3′	
Cav1.2	F: 5′-GACGCTATGGGCTATGAGTTAC-3′	199
	R: 5′-AGTCCAGGTAGCCCTTTAGGT-3′	

**Table II.** ECG data of adult and aged dogs (mean ± SD).

Group	P wave (msec)	PWD interval (msec)	PR interval (msec)	QRS interval (msec)	QT interval (msec)
Adult	66.1±6.4	19.1±4.1	123.9±7.2	63.1±4.3	248.9±11.7
Aged	75.9±5.3^a^	26.7±3.1^a^	130.0±7.7	64.7±5.4	246.5±17.3

a P<0.05 vs. adult canines. ECG, electrocardiogram; PR interval, from starting point of P wave to the starting point of QRS wave in electrocardiogram; QRS interval, rom starting point of QRS wave to the ending point of QRS wave in electrocardiogram; QT interval, from starting point of QRS wave to the ending point of T wave in electrocardiogram.

**Table III.** Action potential characteristics recorded from adult and aged canine atria at a cycle length of 2000 msec (mean ± SD).

Group	MDP (mv)	APA (mv)	Plateau (mv)	APD_90_ (msec)
Adult	−78.8±0.8	109.8±1.4	−6.4±1.1	320.0±7.9
Aged	−79.2±1.4	110.5±4.9	−9.5±1.7	340.5±10.1^a^

a P<0.05 vs. adult canines. MDP, maximum diastolic potential; APA, action potential amplitude; APD_90_, action potential duration to 90% repolarization.

**Table IV.** Electrophysiological characteristics of the L-type calcium current (I_Ca.L_) in adult and aged canine LA cells (mean ± SD).

Group	n	I_Ca.L_ density (pA/pF)	Steady-state activation		Steady-state inactivation		Monoexponential recovery time constants (msec)
	
			V_0.5_ (mV)	K (mV)	V_0.5_ (mV)	K (mV)	
Adult	14	−8.1±0.5	−7.1±1.5	5.7±0.4	−23.1±2.1	6.2±0.3	51.9±3.3
Aged	16	−14.1±0.8^a^	−6.7±2.8	5.5±0.5	−22.9±3.3	6.4±0.5	53.1±3.1

a P<0.05 vs. adult. n, cell number; V_0.5_, half-activation or -inactivation voltage; K, slope factor; pA/pF, current density.
